# Environmental influences on the female epigenome and behavior

**DOI:** 10.1093/eep/dvw007

**Published:** 2016-07-04

**Authors:** Samantha M. Keller, Tania L. Roth

**Affiliations:** ^1^ Department of Psychological and Brain Sciences, University of Delaware, Newark, DE 19716, USA

**Keywords:** environment, epigenetic, female, maternal circuitry, maternal behavior

## Abstract

Environmental factors have long-lasting effects on brain development and behavior. One way experiences are propagated is via epigenetic modifications to the genome. Environmentally driven epigenetic modifications show incredible brain region- and sex-specificity, and many brain regions affected are ones involved in maternal behavior. In rodent models, females are typically the primary caregiver and thus, any environmental factors that modulate the epigenotype of the mother could have consequences for her current and future offspring. Here, we review evidence of the susceptibility of the female epigenome to environmental factors, with a focus on brain regions involved in maternal behavior. Accordingly, implications for interventions that target the mother’s epigenome and parenting behavior are discussed.

## Introduction

Epigenetics, a term coined by Waddington in the 1940s, is used to describe gene–environment interactions that influence phenotype [1]. Epigenetic mechanisms include DNA methylation, histone modifications, and microRNAs (miRNAs), and collectively, afford routes for environmental factors to alter gene activity. DNA methylation refers to the addition of methyl groups onto cytosine residues of a DNA strand. This commonly occurs at dinucleotide cytosine–guanine (CpG) sites, though methylation can also occur at non-CG dinucleotides [2–4]. DNA methylation is catalyzed by a group of enzymes called DNA methyltransferases (DNMTs), of which several types exist. DNMT 1 contributes to the maintenance of DNA methylation by adding methyl groups to hemi-methylated DNA, while DNMT 3a and 3b are able to modulate methylation patterns via *de novo* methylation [5]. Typically, DNA methylation results in the suppression of gene expression; however, under some circumstances it can also enhance gene transcription [6–8].

Posttranslational histone modifications comprise acetylation, methylation, ubiquitylation, sumoylation, and phosphorylation of the N-terminal tail of histone proteins. Because DNA is wrapped around histone molecules within nucleosomes, such modifications can either make DNA more or less accessible for transcription [9–11]. For example, histone acetylation involves the addition of acetyl groups (via histone acetyltransferases) at lysine residues on the N-terminal tail of histone proteins, decreasing the affinity between the histone and DNA and thereby allowing a more permissive transcriptional state [9–11]. Histone deacetylases (HDACs) reverse this process [9–11]. Another mode of epigenetic regulation gaining increasing attention is miRNAs, which are non-coding single stranded RNAs (usually about 22 bp in length) capable of exerting gene silencing effects via degradation or destabilization of mRNA [12–15]. Some studies also indicate that certain miRNAs upregulate gene expression [16, 17].

While epigenetic modifications were once thought to be limited to embryonic development, it has since been discovered that epigenetic modifications in the central nervous system continue to transpire throughout development and into adulthood. Since initial discoveries in the early 2000s, (e.g. [18–22]), investigators have continued to uncover the epigenetic consequences of exposure to various environmental perturbations. Aberrant epigenetic profiles have also been linked to a host of neuropsychiatric disorders [23–26], and epigenetic modifications are increasingly being recognized as important for understanding sex differences in brain development and responses to environmental and psychosocial perturbations. Epigenetic mechanisms are known to mediate sexual differentiation of the brain, and sex differences in DNA methylation resulting from hormonal exposures during the perinatal period are long-lasting and continue to emerge throughout development [27–29]. Indeed, sexually dimorphic DNA methylation is observed at a multitude of genes throughout the genome [30]. However, the role epigenetic mechanisms play in sexually divergent behaviors, such as maternal behavior, is less clear. Males and females are known to differ in prevalence rates across a multitude of psychiatric disorders [31] and it has been proposed sex differences in the epigenome contribute to this disparity [32]. As female subjects are certainly underrepresented in behavioral neuroscience literature [33, 34], and because experiences altering the brain and behavior of females have implications for future generations due to the critical roles of infant–mother interactions and the quality of maternal care in offspring development, we chose to focus this review on data acquired from female subjects.

## Overview of Rodent Maternal Behavior and Circuitry

**Figure 1:**
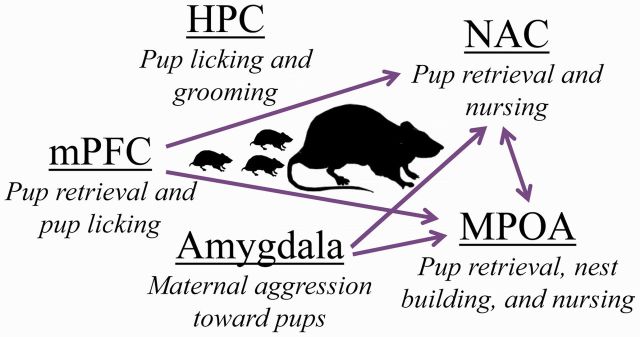
Neuroanatomy underlying maternal behavior. This figure illustrates several of the key neuroanatomical regions underlying maternal behavior which are subject to epigenetic modulation by environmental factors as described in this review. Several maternal behaviors each region has been implicated in are also listed. The arrows indicate a simplified neuroanatomical circuit of projections amongst brain regions [35, 57, 150]. Abbreviations: HPC = hippocampus; NAC = nucleus accumbens; mPFC = medial prefrontal cortex; MPOA = medial preoptic area

Before delving into the epigenetics literature, here we mention several maternal behaviors and neuroanatomical substrates that are discussed in various sections of the review. For a more thorough evaluation of these topics, we refer the reader to several excellent reviews (e.g. [35–38]). One of the predominant maternal behaviors observed in laboratory rodents is licking of the pup’s body, with an emphasis on the anogenital area (anogenital licking aids in waste elimination) [39, 40]. Mothers spend a significant amount of time in the nest hovering over pups, engaging in bouts of licking, and nursing [40, 41]. Retrieval of pups becomes necessary as they wander from the nest, and this maternal behavior is elicited by ultrasonic vocalizations emitted by pups [42]. Of note, nulliparous females display retrieval behavior after continuous exposure (sensitization) to pups [43, 44]. Further, dams will engage in a behavior referred to as tail chasing, in which a dam chases their tail, eventually picking it up and carrying it in her mouth [45, 46]. The specific function of tail chasing is not known, but may be related to pup retrieval as dams often engage in this behavior antepartum and outside of the nest area, carrying the tail back to the nest [45]. Finally, brain regions involved in maternal behavior include the bed nucleus of the stria terminalis (BNST) [47–49], paraventricular nucleus (PVN) [50–52], nucleus accumbens [53–55], prefrontal cortex (PFC) [56–58], medial preoptic area (MPOA) [22, 57, 59–62], amygdala [60, 63, 64], and hippocampus [65, 66]. Several of these regions and their role in regards to maternal behavior are depicted in Fig. 1.

## Adulthood and Preconception Psychosocial Stress

Stressors experienced in adulthood are capable of modulating the female epigenome and behavior. In one study that implemented a chronic variable mild stress paradigm, adult female rats were found to have increased levels of the histone acetyltransferase cyclic AMP response element-binding protein (CBP) in the BNST [67]. This effect was not seen in male rats [67]. These data suggest an important role of histone acetylation in response to stress exposure that could lead to sex-specific alterations in behavioral outcomes. As further evidence for this notion, in an acute restraint stress paradigm that elicited elevated corticosterone and corticotropin releasing factor (*Crf*) mRNA in the PVN in male but not female rats, males demonstrated elevated CBP levels and females did not [68]. In another study that employed a subchronic variable stress paradigm to produce a depression-like phenotype, female mice had increased levels of *Dnmt3a* within the nucleus accumbens [69]. Mice with a knock-out of *Dnmt3a* in the nucleus accumbens showed resilience to the subchronic variable stress, providing further support for the concept that *Dnmt3a* overexpression might mediate stress-induced depression [69]. Taken together, these studies show that the female brain can be epigenetically modulated in key components of maternal behavior circuitry by stress exposure. Further research is needed to understand the functional significance of sex differences in these epigenomic marks induced by these stressors.

Stress incurred by a female prior to pregnancy is also capable of modulating brain and behavioral trajectories of her offspring. In adult female rats that underwent a 7-day chronic unpredictable stress regimen, corticotrophin releasing factor receptor type 1 (*Crf1*) mRNA was upregulated in the ova and frontal cortex [70]. A separate group of females were bred 2 weeks after termination of the same stress paradigm, and their offspring were likewise found to have increased *Crf1* expression in their brain in both infancy (on postnatal day 0, prior to any maternal care received) and adulthood [70]. This altered gene expression corresponded with behavioral alterations when offspring were adults, including potentiated startle responses and increased locomotor activity in the elevated plus maze [70]. Preconception-stress-exposed rats and their first-generation female offspring also showed increased corticosterone levels. In contrast, second-generation offspring showed reduced expression of *Crf1* mRNA and decreased corticosterone levels [71]. These data provide evidence that a stressor encountered by an adult female can contribute to the programming of HPA-axis reactivity for several generations.

While stressful experiences can modulate the epigenome and introduce maladaptive behavioral outcomes, other types of experiences can exert adaptive influences on behavior through epigenetic mechanisms. Induction and maintenance of maternal behavior in response to pup interaction has been proposed to result from experience-driven chromatin remodeling [72]. In a maternal sensitization paradigm, which involved repeatedly introducing virgin nulliparous female mice to pups to stimulate maternal behavior, histone acetylation was shown to be a critical mediator for this experience-induced behavioral change [43]. Administration of the HDAC inhibitor (HDACi) sodium butyrate reduced the amount of time required for a nulliparous female to display maternal care toward pups [43]. This pharmacological manipulation also increased gene expression of estrogen receptor β, CBP, and the oxytocin receptor in the MPOA [43]. Furthermore, the HDACi-induced facilitation of maternal behavior and gene expression lasted for a month after initial maternal experience [73]. Taken together, these studies provide evidence that the induction of maternal behavior has epigenetic underpinnings and that administration of certain epigenome modifying drugs can have long-term facilitatory effects on maternal responsiveness.

## Gestational Stressors

Epigenetic mechanisms also provide routes through which gestational stressors, either psychosocial or chemical in nature, can affect offspring. For example, prenatal predator exposure is one stressor that has both epigenetic and behavioral consequences. Female adult offspring of pregnant mouse dams exposed to predator odor demonstrated an enhanced corticosterone response and an increase in anti-predator behaviors [8]. This behavioral profile corresponded with increased *Crf1* mRNA in the amygdala and decreased Brain-derived neurotrophic factor (*Bdnf*) mRNA and DNA methylation of *Bdnf* exon IV in the hippocampus [8]. Daily exposure to restraint stress during pregnancy similarly modulates the epigenetic profile and levels of epigenetic regulators in rat offspring. The placenta of fetal offspring exposed to this gestational stress had increased levels of *Dnmt3a* mRNA and enhanced methylation of the 11β-hydroxysteroid dehydrogenase type 2 (*Hsd11b2*) gene promoter [74]. These same animals also displayed reduced levels of CpG methylation within the *Hsd11b2* promoter region and increased methylation at sites within exon 1 of the hypothalamus as well as enhanced *Dnmt1* mRNA within the cortex [74]. Further, adult female offspring of mouse mothers exposed to chronic unpredictable stress during gestation demonstrated impaired spatial memory capabilities, higher plasma corticosterone levels, decreased levels of H3 acetylation, and increased DNMT1 protein in the hippocampus [75].

miRNAs have been gaining attention for their ability to influence gene activity, though limited work has examined miRNAs in the female brain [76]. Rat dams exposed to stress (restraint and forced swim) during pregnancy demonstrated a decrease in the incidence of tail chasing, and a correlational upregulation of 147 miRNAs and downregulation of 195 miRNAs in their frontal cortex [77]. Target genes of the affected miRNAs had roles in hormonal regulation, brain pathologies, stress responsivity, and neurotransmission [77]. While a similar profile of altered microRNA expression was found in the brains of their male offspring, future examination is required to determine if female offspring would likewise show disrupted miRNA profiles.

Chemical perturbations during gestation likewise affect female offspring [78, 79]. Bisphenol A (BPA) is an endocrine disrupting chemical gaining increasing attention for its widespread use and association with the development of diseases [80–83]. BPA is of particular concern for females due to its ability to modulate estrogen and alter epigenetic profiles [84, 85]. Female offspring exposed to BPA, either during gestation alone or during both gestation and early postnatal development grew up to spend less time performing nurturing maternal behaviors toward their own offspring, and similarly adult females administered BPA demonstrated fewer maternal behaviors toward their offspring [86–88]. BPA exposure also modulates levels of epigenetic regulators within brain regions involved in maternal behavior, which could underlie the observed deficits in maternal behavior in BPA-exposed females. Specifically, levels of DNMT1 and DNMT3a were altered within the hypothalamus and PFC of juvenile female mice prenatally exposed to BPA [88]. Gestational and early postnatal exposure of rats to endocrine disrupting chemicals including estradiol benzoate and methoxychlor resulted in elevated estrogen receptor (*ERα*) mRNA and increased DNA methylation in the POA [89]. In adulthood, these perinatally exposed animals also experienced the advancement of reproductive senescence [89].

While antidepressant drugs mitigate depressive-like behavior in adult animals, developmental antidepressant exposure can have deleterious effects [90, 91]. Adult rat females that were prenatally exposed to fluoxetine displayed enhanced depression-like behavior, as assessed via the forced swim test [90]. Changes in the hippocampus of these females included decreased *Bdnf* exon IV mRNA and increased histone 3 lysine 27 trimethylation [90]. The presence of *Bdnf* mRNA was negatively correlated with immobility time in the forced swim test, suggesting that the observed epigenetic profile in these animals contributed to the phenotypic outcomes associated with developmental fluoxetine exposure [90]. Taken together, data highlighted in this section illustrate that gestational perturbations certainly have influences on neurobiological and behavioral outcomes in female offspring.

## Rearing Environments

Rearing environments of rodent pups have long been recognized for their profound influence on the development of behavior, including maternal behavior [40, 92–95]. Female Long-Evans rats demonstrate a natural variability in their quality of maternal care, with some females exhibiting high levels of licking/grooming (LG), and others displaying low levels of LG [96]. This variability in maternal care is generationally transmitted, as female rats that were exposed (either born or cross-fostered) to a low-licking and grooming mother in their infancy demonstrate low levels of LG toward their own offspring [97, 98]. Work utilizing natural variations in LG maternal behavior found epigenetic modulation of the *ERα* gene within the MPOA of dams. Specifically, low-LG mothers showed decreased *ERα* gene expression within the MPOA relative to high-LG mothers [22]. This effect was transmitted to female offspring, but cross-fostering these offspring with a high-LG mother rescued *ERα* expression, showing that mother–infant interactions early in life are critical for MPOA development [99]. Social enrichment postweaning also enhanced LG behaviors in low-LG female offspring [100]. The variability of *ERα* expression and transmission of LG behaviors to offspring is mediated by DNA methylation, as low-LG caregivers demonstrate higher methylation of the *ERα1b* promoter [22]. The MPOA is a sexually dimorphic region critical in maternal behavior [22, 57, 59–62] and estrogen is a transcription factor with known protective effects [84]. Estrogen interacts with histone acetylation, suggesting a route for estrogen to affect expression of many genes [85, 101]. Estrogen levels also affect sexual behaviors, allowing for epigenetic alterations to have effects on mating capabilities of females [101, 102].

It is well established that the maternal behavior directed toward male versus female offspring differs, as dams spend more time licking their male pups than their female pups [39, 103, 104]. Because of the sex-specific nature of maternal care, altering the sex composition of litters changes pup-directed maternal behavior [105]. The resulting alterations in maternal behavior have lifelong effects on the brain and behavior of these offspring [106–108]. Within the *Oprm1* gene promoter, which encodes for the µ-opioid receptor, it was discovered that female rats raised in female-only litters demonstrated higher levels of methylation within the hippocampus as compared with females who belonged to mixed litters (i.e. litters containing both male and female offspring) [109]. No effects on DNA methylation of *Oprm1*were found within the nucleus accumbens, suggesting that this effect is brain-region specific [109]. The µ-opioid receptor is critical for mother–infant relationships, and thus, modulation of this receptor within attachment and maternal behavior circuitry could have critical implications for the maternal behavior of these offspring [110]. Another study that manipulated litter sex composition found hypermethylation of the hippocampal *GR* gene in adolescent female rats from female-only litters [111]. Because females receive less LG than their male counterparts, this corroborates other data with regard to lower LG behavior and enhanced DNA methylation of the *GR* gene in offspring [18].

**Figure 2:**
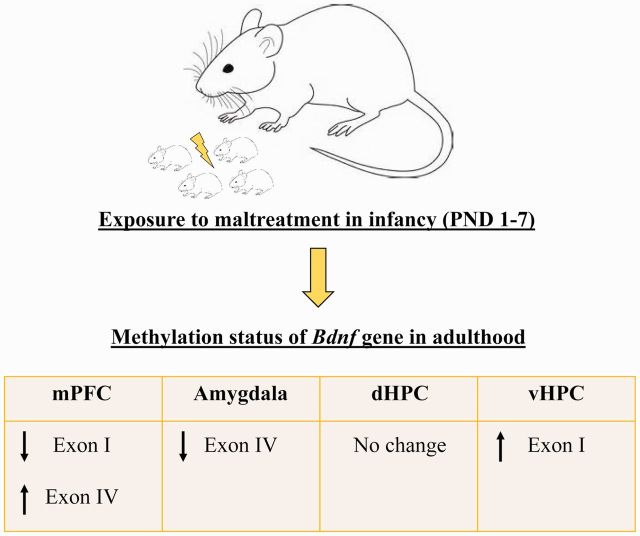
Epigenetic modulation of the *Bdnf* gene by early-life stress. Exposure to caregiver maltreatment in infancy has lifelong implications on the methylation status of the *Bdnf* gene. This figure summarizes the changes in DNA methylation of exons I and IV which are present in adulthood in female animals that were exposed to caregiver maltreatment in the first week of life. Abbreviations: mPFC = medial prefrontal cortex; dHPC = dorsal hippocampus; vHPC = ventral hippocampus; PND = postnatal day

Our lab and others have studied the effects of aversive rearing experiences on the Brain-derived neurotrophic factor (*Bdnf*) gene (Fig. 2). The medial prefrontal cortex (mPFC) is a region critical for cognitive and memory processes and has been implicated in several neuropsychiatric disorders [112–116]. In addition, lesions to the mPFC disrupt maternal behaviors such as pup retrieval and pup licking [56, 58]. In a model of early-life maltreatment whereby rat pups are exposed to 30-min bouts of caregiver maltreatment (frequent stepping on, dropping, dragging, actively avoiding and rough handling) daily for the first postnatal week, variability in gene expression and DNA methylation can be detected in developing and adult females [117–120], and further, maltreated-females grow up to mistreat their own offspring [119].

Within the whole PFC, DNA methylation of the *Bdnf* gene was enhanced across the lifespan (during infant, adolescent, and adult time points) in maltreated animals, which corresponded with decreased *Bdnf* expression in adult females [119]. Within the mPFC, female pups subjected to maltreatment displayed a transient decrease in DNA methylation at the *Reelin* gene, which was no longer present in adolescence or adulthood [117]. However, these females showed decreased gene expression of *Reelin* in adulthood, signifying that although DNA methylation was no longer different, these developmental experiences resulted in differential expression of *Reelin* [117]. Adult females also displayed decreased methylation of *Bdnf* exon I but increased methylation of *Bdnf* exon IV in adulthood [117]. Gadd45b, which plays a role in DNA demethylation [121, 122], was the only epigenetic regulator significantly altered (lower mRNA levels) within the mPFC of adult females [123], thus the mechanism (or mechanisms) underlying the maltreatment-induced alterations in female gene expression and DNA methylation remains to be elucidated.

Using the same maltreatment regimen, female-specific modulations were also detected within the amygdala. The amygdala is a region involved in maternal behavior [60, 63, 64] and amygdalar pathways are particularly involved in maternal aggression [124–126]. Female rats that were maltreated in infancy displayed reduced expression of the oxytocin receptor gene in infancy and adolescence [127], a gene important for maternal behavior (i.e. higher oxytocin receptor levels are associated with more maternally responsive females) [99, 128, 129]. During adolescence, enhanced DNA methylation of the *Bdnf* gene [118], and decreased *Bdnf* gene expression and increased Neuropeptide Y (*NPY*) gene expression [127] were found. Contrary to adolescent gene expression, *Bdnf* gene expression was enhanced in females in adulthood [127] and this paralleled lower methylation levels [120]. These results further illustrate the transient and dynamic nature of epigenetic changes resulting from caregiver experiences. It is currently unclear what mechanism (or mechanisms) could underlie these changes. To further probe these effects of early-life experience and ascertain the way by which these epigenetic modifications could alter maternal behavioral outcomes, an important factor in future research will be parsing apart the nuclei within the amygdala that are functionally distinct and differentially contribute to maternal behavior [64].

Finally, restricted access to a caregiver can also have long-lasting epigenetic and behavioral consequences for female offspring [130]. In a mouse model of maternal separation, adolescent female C57BL/6J mice that experienced daily separation from their mother from postnatal days (PND) 1 through PND 14 demonstrated decreased *Bdnf* gene expression and enhanced *GR* methylation in the hippocampus [131]. Interestingly, in Balb/cJ mice that were exposed to this same manipulation, *Bdnf* expression was enhanced within the PFC and increased levels of *Bdnf* exon IX methylation within the hippocampus were found [131]. Altogether, data highlight the brain region-dependent nature of epigenetic modifications in females in response to different rearing environments.

## Generational Transmission of Epigenetic Modifications and Phenotypes

Quality of maternal behavior is passed from mother to female offspring [18, 22, 96, 97, 119, 128]. The transmission of LG behaviors from mother to female offspring is mediated by rearing experience, as cross-fostering offspring to high-LG mothers is sufficient to enhance LG levels [18, 97]. In addition, LG behaviors are enhanced by social experiences post-weaning [100]. In a multigenerational stress design where three generations of rats were exposed to restraint and swim stressors, changes in antepartum behavior were found to be altered across generations [45]. Specifically, tail chasing behavior prior to parturition varied as a consequence of multiple generations of stress exposure. The first generation exposed to the stressor did not show behavioral variations, however, the second and third generations of stress-exposed females showed a reduction in tail chasing behavior, with the third generation showing the most severe decrease in tail chasing prevalence [45]. In addition to programming of antepartum and maternal behavior, stress responsivity is likewise transmitted from parent to offspring [70, 71].

Another line of generational transmission comes from studies in rats using the endocrine disruptor vinclozolin, which is known to produce pregnancy abnormalities and kidney disease [132]. In addition, vinclozolin when limited to F0 exposure and then using male offspring to generate successive generations, alters expression levels of over 1000 hippocampal and 100 amygdala genes in F3 generation females, with concomitant increases in anxiety-like behavior [133]. In our own research where we found enhanced levels of DNA methylation of the *Bdnf* gene in the PFC and hippocampus in female rats with a history of maltreatment, we found this same change in the next generation of offspring [119]. Interestingly, cross-fostering pups of dams that experienced maltreatment in infancy was not sufficient to completely rescue DNA methylation levels [119]. This might indicate that these epigenetic marks were heritable (i.e. the associated epigenetic marks were transmitted through the germline as a result of environmental experiences). Prepartum behavior however was different in females that had been exposed to maltreatment such that previously maltreated dams displayed more anxiety-related behaviors during the last 3 days of pregnancy [119]. Thus, it is uncertain whether the biological effects ascertained in our model were due to a compromised gestational environment (i.e. maternal state during gestation) or that the epigenetic marks were passed through the germline. Regardless of the mode of transmission in our study or others highlighted here, together data indicate that stress exposure in females has behavioral and epigenetic consequences for her offspring and grand-offspring. More research is certainly warranted in the areas of behaviorally mediated vs. germ-line-mediated inheritance.

## Interventions to Alter the Female Epigenome

The inherently malleable epigenome may be a target of therapeutic or behavioral intervention, and many studies have shown this to be true (e.g. [119, 134, 135]). However, sex differences exist under basal conditions in levels of various epigenetic regulators, and these sex differences in epigenetic regulators contribute to sex differences in behavior (e.g. [29, 136]). For example, within the amygdala, mRNA levels of *Dnmt3a, MeCP2* [137], and Gadd45b [138] are higher in developing females as compared with males. Sex differences are also found in baseline levels of posttranslational histone modifications and DNA methylation throughout other regions of the brain including the cortex, hypothalamus, and BNST/POA [139–141]. This suggests that experiments manipulating these molecules may see divergent effects between the sexes. In addition, levels of these regulators are dynamic and levels between the sexes differ across developmental time periods, so assessing the efficacy of administration of epigenetic regulators across the lifespan is of importance [142].

There are some data to support the notion that drugs that manipulate the epigenome can have positive effects on the female brain. For example, administration of a DNMT inhibitor in adulthood rescued aberrant PFC DNA methylation patterns resulting from exposure to caregiver maltreatment [119]. This suggests that modulating DNA methylation profiles could be utilized to normalize consequences of early-life stress, even when the intervention occurs in adulthood. Similarly, epigenetic modifications resulting from inhibition of HDACs have beneficial effects, although very few of the studies that have been conducted included female subjects. Sodium butyrate decreased depressive-like behaviors in mice, and this effect was further enhanced by co-administration with the antidepressant fluoxetine [91]. In a rat model of neonatal maternal separation, adult females exposed to separation from PND2-9 demonstrated a reduced fear-potentiated startle response, which corresponded with increased serum estradiol and decreased histone methylation in the frontal cortex [143]. Treatment with the HDACi valproic acid, but not the DNMT inhibitor 5-aza-2′-deoxycytidine, prior to daily maternal separation reversed this decrease in fear-potentiated startle behavior and histone methylation [143]. As the DNMT and HDAC inhibitors employed in these studies lack target specificity (i.e. many gene loci would be presumed to be affected) and can produce off-target effects, there is a strong need to explore strategies that enable select epigenetic modifications.

Maternal diet has strong modulatory effects on the epigenome, health, and behavioral outcomes of her offspring. Supplementing maternal diet with folic acid, a methyl donor, ameliorated aberrant epigenetic profiles in mouse offspring induced by exposure to BPA [78]. Methyl donor supplementation also rescued alterations in DNA methylation and behavior resulting from exposure to high fat diet during gestation. Specifically, when a high fat diet was paired with methyl donor supplementation, the global hypomethylation typically induced by gestational exposure to high fat diet was eliminated in the PFC of female rat offspring [144]. This treatment also ameliorated the enhancement of µ-opioid receptor mRNA in the nucleus accumbens and PFC, showing dietary supplementation is capable of rescuing both global and gene-specific aberrations induced by gestational exposure to high fat diet [144]. Further, methyl donor supplementation rescued the high-fat diet preference and reduced locomotor activity observed in offspring of dams that consumed a high-fat diet during pregnancy [144]. Such epigenetic alterations could contribute to differences in processing rewarding stimuli. Because maternal behavior is a motivated behavior and pup-interactions elicit a reward response in dams [145–147], aberrant reward processing could contribute to deficient maternal behavior toward offspring. Taken together, these data highlight the ability of the maternal diet to regulate the epigenome of offspring and the therapeutic potential for dietary supplementation during the prenatal/early postnatal period.

Environmental interventions similarly rescue LG behavior in females that received low levels of LG in infancy. As previously mentioned, social enrichment post-weaning enhances levels of LG behavior in females that received low LG in infancy. This coincided with enhanced oxytocin receptor binding and exploratory behavior as measured by the open-field test [100]. This suggests that social interactions for the female beyond those occurring in infancy have behavioral and neurobiological implications, and such manipulations are capable of modulating behavioral trajectories. Natural variation in maternal care also has implications for the development of reward systems, with adult offspring of low-LG dams exhibiting a blunted increase in the dopamine signal within the nucleus accumbens in response to licking and grooming pups. Administration of the selective dopamine re-uptake inhibitor GBR 12909 brought the dopamine signal generated by pup interactions up to that of high-LG offspring [55]. Thus, drugs that manipulate dopamine systems could have positive effects for maternal care-associated deficits in reward processing, and future studies could examine potential epigenetic underpinnings of this drug action and efficacy.

## Concluding Remarks

We have highlighted data demonstrating that environmental factors throughout development modify the female epigenome and create epigenetic marks within brain regions critical for maternal behavior. Epigenetic marks can be both long-lasting and dynamic (i.e. some continue to transpire over the course of the lifespan or can be modified). Future research is needed to better ascertain the effects of environmental and psychosocial perturbations on the female epigenome, as well as motherhood itself. These data are important to have in hand, as their understanding has implications for interventions for negligent/abusive maternal care as well as for neuropsychiatric disorders such as postpartum depression, which is estimated to occur in about 15% of pregnancies and could potentially have epigenetic underpinnings [148, 149].

## Funding

This study was supported by a grant from The National Institute of General Medical Sciences (1P20GM103653).

*Conflict of interest statement*. None declared.
